# One-year all-cause mortality and comorbidity predictors in 14,975 adults with PCR-confirmed COVID-19: a retrospective Turkish cohort study

**DOI:** 10.7717/peerj.21206

**Published:** 2026-04-20

**Authors:** Recep Balık, Nurgül Ceran, Seniha Şenbayrak, Asuman İnan, Serpil Erol, Yelda Balık, Aytekin Kaymakcı, Tolga Katmer, Abdullah İbrahim

**Affiliations:** 1 Department of Infectious Diseases and Clinical Microbiology, University of Health Sciences Haydarpaşa Numune Training and Education Hospital, İstanbul, Turkey; 2 Department of Intensive Care Unit, University of Health Sciences Haydarpaşa Numune Training and Research Hospital, İstanbul, Turkey; 3 Department of Pediatric Surgery, University of Health Sciences Ümraniye Training and Research Hospital, İstanbul, Turkey; 4 Vice President of Public Hospitals Services, Muğla Provincal Healts Directorate, Muğla, Turkey; 5 Department of Emergency Medicine, University of Health Sciences Haydarpaşa Numune Training and Research Hospital, İstanbul, Turkey

**Keywords:** COVID-19, One-year mortality, Risk factors, Comorbidities

## Abstract

**Background:**

Coronavirus disease 2019 (COVID-19), caused by severe acute respiratory syndrome coronavirus 2 (SARS-CoV-2), produces outcomes ranging from mild illness to death. Although in-hospital prognostic factors are well described, long-term mortality data are scarce, particularly from middle-income countries with relatively young populations. We therefore quantified one-year all-cause mortality and identified independent predictors in a large Turkish cohort support follow-up planning and prevention for high-risk groups.

**Methods:**

We retrospectively included all adults (≥18 years) with polymerase chain reaction–confirmed SARS-CoV-2 infection who presented to Haydarpaşa Numune Training and Research Hospital between 1 March 2020 and 31 January 2021, covering the pre-vaccination period in Türkiye. Demographics, smoking status, and ICD-10 coded comorbidities were extracted from hospital’s electronic medical record system and national electronic health database. Follow-up was complete: 98.9% had 365 days; the remaining 168 had 360–364 days and were administratively censored, with no losses to follow-up. Missing smoking data was imputed by multiple imputations with chained equations. One-year survival status was obtained from the death registry. Risk factors were examined by Cox proportional hazards models after verifying assumptions.

**Results:**

Among 14,975 patients (median age 40 years, interquartile range 28.5–52.2; 50.8% male), 357 deaths occurred within 365 days, giving one-year mortality of 2.4% (95% CI [2.1–2.6]%). Non-survivors were older (71.8 *vs* 39.5 years), more often male (63% *vs* 50.5%), and had higher rates of type 2 diabetes mellitus, ischemic heart disease, heart failure, chronic kidney disease, and cancer. In multivariable analysis each additional year of age increased risk by 10%. Other independent predictors were male sex, type 2 diabetes mellitus, ischemic heart disease, heart failure, chronic kidney disease, and cancer. Smoking was inversely associated with mortality after inverse-probability weighting. Model discrimination was excellent. Only cancer showed minor time-dependency, mainly within the first 60 days of follow-up; two-thirds of cancer-related deaths occurred by day 60. Bootstrap optimism correction confirmed robustness.

**Conclusion:**

One-year mortality after COVID-19 in this relatively young Turkish population was low yet clinically meaningful. Persistent excess risk among older adults, men, and patients with cancer or major cardiometabolic disease supports structured post-acute surveillance, prioritized vaccination boosters, and aggressive management of underlying conditions. The inverse association with smoking is probably due to residual or unmeasured confounding and should not influence practice. These findings refine long-term risk stratification and can guide resource allocation as COVID-19 becomes an endemic threat external validation that incorporates vaccination status and viral variants is warranted.

## Introduction

After the first case of coronavirus disease 2019 (COVID-19), caused by severe acute respiratory syndrome coronavirus 2 (SARS-CoV-2), was reported in Wuhan, China in December 2019, over 270 million cases had been documented globally by December 2021 (WHO, 2021). Within three months, the World Health Organization declared COVID-19 a pandemic. COVID-19 presents a broad clinical spectrum, ranging from mild illness to death (CDC, 2020). Several comorbid conditions, such as cancer, chronic kidney disease (CKD), chronic lung disease (CLD), type 2 diabetes mellitus (T2DM), and cardiovascular disease, have been associated with a higher risk of severe COVID-19 outcomes (Goel et al., 2021). Previous studies have explored risk factors for mortality that vary according to geographic location and population characteristics (Ioannou et al., 2020; Chatterjee et al., 2021). Although a growing body of research has demonstrated associations between comorbidities and mortality, large-scale studies with extended follow-up periods remain limited (Chatterjee et al., 2021). In this study, we aimed to quantify 1-year all-cause mortality and to identify independent predictors of 1-year mortality in a large Turkish cohort with PCR-confirmed COVID-19.

## Materials & Methods

**Figure 1:**
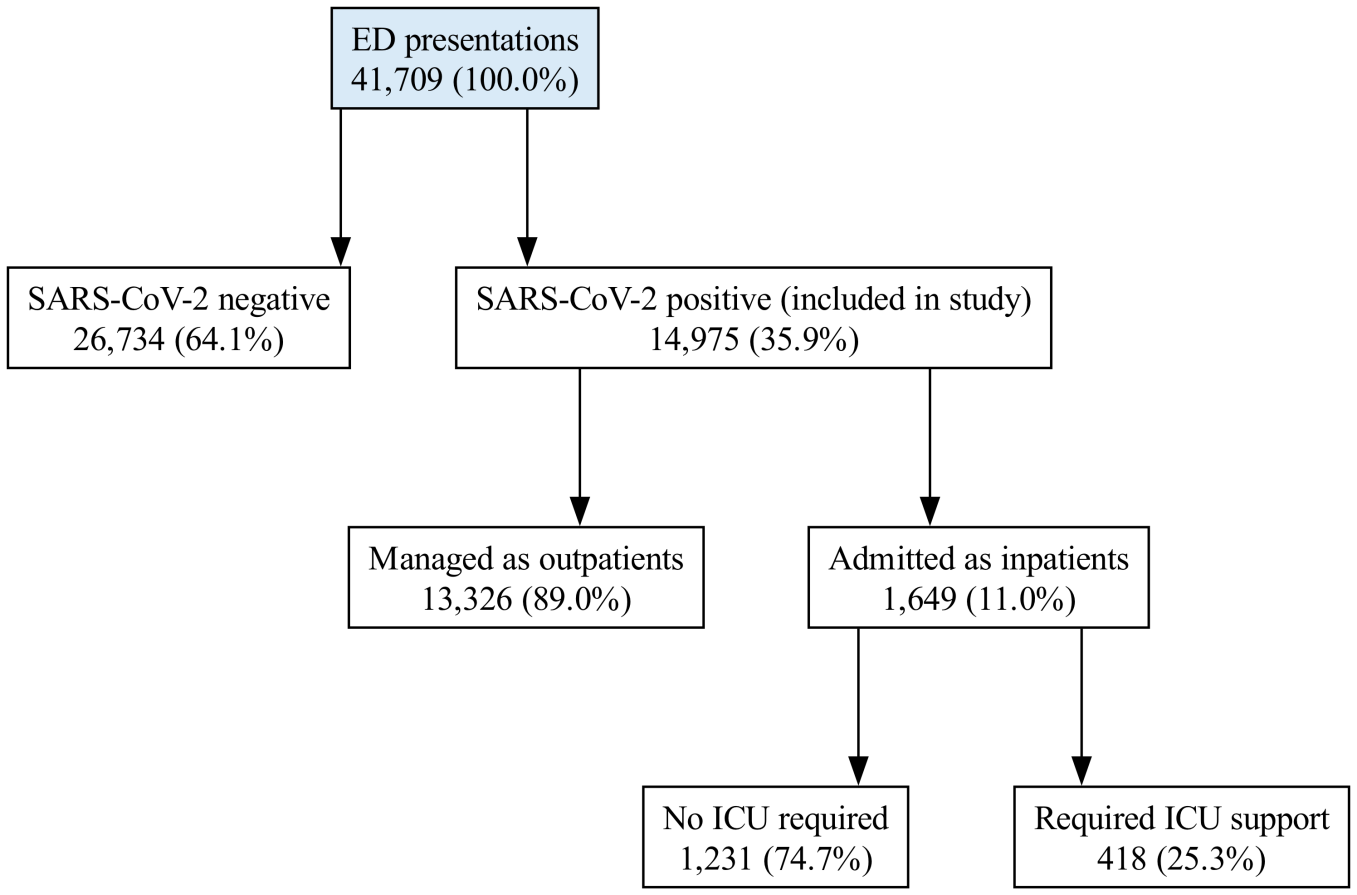
Flowchart of patient selections.

This retrospective observational study was conducted in accordance with the Declaration of Helsinki after obtaining approval from the institutional ethics committee (Haydarpaşa Numune Training and Research Hospital Ethical Committee, Approval reference number: 2021-KAEK-47). Adult patients (aged 18 years and older) who presented to the emergency department of Haydarpaşa Numune Training and Research Hospital with a COVID-19–like illness and had SARS-CoV-2 infection confirmed by polymerase chain reaction (PCR) testing of nasopharyngeal or oropharyngeal swab specimens obtained at the time of emergency department presentation between March 2020 and January 2021 were retrospectively included (Fig. 1). This cohort was assembled before widespread COVID-19 vaccination in Türkiye.

 The study was conducted at a tertiary-care teaching hospital that serves as both a referral center and a first point of contact through its emergency department. During the pandemic period, the hospital functioned as a designated COVID-19 referral center, receiving patients from across the metropolitan region through both walk-in emergency admissions and referrals from community hospitals.

Demographic characteristics, comorbidities, and smoking status were obtained from both the hospital’s electronic medical record system and national electronic health databases. Comorbidities were identified using International Classification of Diseases, Tenth Revision (ICD-10) codes recorded in either data source. Specific ICD-10 codes corresponding to each condition are listed in Table S4. Smoking status was derived from structured registry fields and defined as active smoking at the time of SARS-CoV-2 diagnosis, referring to regular (daily or habitual) tobacco use documented in either the hospital or national record. Former or occasional smokers were classified as non-smokers for the purpose of analysis. Mortality outcomes were verified through linkage with the national death notification system. Cross-referencing these datasets using unique patient identifiers ensured high completeness and internal consistency. All-cause 1-year mortality was defined as either in-hospital death or out-of-hospital death occurring within 365 days following the first positive SARS-CoV-2 PCR test. Patients were followed from the date of the first positive SARS-CoV-2 PCR test until death or censoring. Follow-up was nearly complete: 98.9% had 365 days of observation. The remaining 168 had 360–364 days of follow-up and were administratively censored at the last available registry date; there were no losses to follow-up.

Demographic characteristics and comorbid conditions were extracted from electronic medical records, with comorbidities identified using ICD-10 codes. To prevent immortal time bias, smoking status was required to be recorded on the same day as the first positive PCR test. Smoking data were missing in 3.75% of patients. These missing values were imputed using multiple imputations by chained equations (MICE), with ten iterations and ten imputed datasets. Imputed datasets were analyzed separately, and Rubin’s rules were used to pool parameter estimates and standard errors (Van Buuren & Groothuis-Oudshoorn, 2011). Patient data were anonymized by assigning unique case identifiers. Out-of-hospital deaths were obtained from the national public health management system.

Continuous variables were presented as mean ± standard deviation (SD) or median with interquartile ranges (IQR), as appropriate. Categorical variables were expressed as counts and percentages. The Anderson-Darling test was used to assess normality due to the large sample size. Normally distributed variables were compared using independent-sample t-tests; otherwise, the Mann–Whitney U test was used. Categorical variables were analyzed using chi-square tests or exact tests when expected cell counts were low. Survival curves were compared using the log-rank test. Mortality risk factors were assessed using univariable and multivariable Cox proportional hazards models. The multivariable model included all clinically relevant variables: age (continuous), sex, smoking status, and nine comorbidities (hypertension, T2DM, ischemic heart disease, heart failure, chronic kidney disease, chronic lung disease, liver disease, obesity, and cancer). The proportional hazards assumption was evaluated using Schoenfeld residual plots.

To assess whether the association between age and 1-year mortality was linear, a Cox model with restricted cubic splines (four knots) was fitted. Propensity scores were estimated from age, sex, smoking status, T2DM, hypertension (HT), heart failure (HF), ischemic heart disease (IHD), liver disease (LD), CLD, obesity, CKD, and cancer. These propensity scores were used to construct stabilized inverse probability weights (IPW), which re-weight the sample to create a pseudo-population where measured baseline covariates are more comparable between the groups being contrasted, thereby reducing confounding from these observed factors. In a secondary analysis restricted to hospitalized patients, in-hospital mortality was modeled using Fine–Gray subdistribution hazard regression, treating discharge alive as a competing event. In competing-risks settings, a competing event prevents the event of interest (in-hospital death) from occurring during hospitalization; the Fine–Gray model estimates covariate effects on the cumulative incidence of in-hospital death while accounting for this competing risk. Multicollinearity was assessed by calculating variance inflation factors (VIFs) in the multivariable Cox model, with a VIF >5 considered problematic. The proportional hazards assumption was further evaluated using scaled Schoenfeld residuals, with both individual-term and global chi-square (*χ*^2^) tests reported. Internal validity was assessed using 200-iteration bootstrap optimism correction and 10-fold cross-validation. All statistical analyses were performed using R version 4.1.1 (https://cran.r-project.org/).

## Results

We included 14,975 adults with PCR-confirmed COVID-19 between March 2020 and January 2021. The median age of the overall cohort was 40 years, and 50.8% were male. Active smoking was reported in 34.6% of patients. Common comorbidities included HT (17.5%), T2DM (17.3%), CLD (8.6%), IHD (8%), LD (4.8%), cancer (4.2%), CKD (3.3%), HF (3.3%), and obesity (2.6%). Overall mortality was 2.4% (Figs. S1–S2). Non-survivors were significantly older than survivors. The proportion of males was higher among non-survivors than survivors. Smoking was significantly less common among non-survivors compared to survivors. The prevalence of comorbidities was significantly higher among non-survivors, including HT, T2DM, CLD, IHD, cancer, CKD, and HF. No significant differences were observed for LD and obesity. Patient characteristics stratified by survival status are presented in Table 1.

**Table 1:** Patient characteristics by survival status.

Variable	Survivors (*n* = 14,618)	Non-Survivors (*n* = 357)	*p*-value
Age (years)	39.5 (28.3–51.1)	71.8 (63.2–80.8)	<0.001
Gender			<0.001
Male	7,388 (50.5%)	225 (63%)	
Female	7,230 (49.5%)	132 (37%)	
Smoking	5,124 (35.1%)	58 (16.2%)	<0.001
Hypertension	2,389 (16.3%)	235 (65.8%)	<0.001
Type 2 diabetes mellitus	2,415 (16.5%)	182 (51%)	<0.001
Chronic ung misease	1,194 (8.2%)	88 (24.6%)	<0.001
Ischemic heart disease	1,028 (7%)	164 (45.9%)	<0.001
Liver disease	705 (4.8%)	20 (5.6%)	0.58
Cancer	530 (3.6%)	97 (27.2%)	<0.001
Chronic kidney disease	412 (2.8%)	86 (24.1%)	<0.001
Heart failure	401 (2.7%)	86 (24.1%)	<0.001
Obesity	376 (2.6%)	7 (2%)	0.58

**Notes.**

Values are presented as number (percentage), age is reported as median (interquartile range).

Among those who were hospitalized (*n* = 1,649), the median age was 58.2 years, and 54.9% were male. Active smoking was reported in 18.4% of hospitalized patients. HT was the most common comorbidity, followed by T2DM, IHD, CLD, cancer, HF, CKD, LD, and obesity. Intensive care unit admission occurred in 25.3%, and 15.3% required intubation. Mortality was observed in 18.9% of hospitalized patients. Hospitalized non-survivors were older than survivors, and a higher proportion were male. Non-survivors had a significantly higher prevalence of HT, T2DM, IHD, CLD, HF, CKD, and cancer. No significant differences were found for smoking, LD, and obesity. Table 2 summarizes these characteristics of hospitalized patients.

**Table 2:** Characteristics of hospitalized patients by survival status.

Variable	Survivors (*n* = 1,338)	Non-Survivors (*n* = 311)	*pa*-value
Age (years)	55.9 (45.8–65.3)	73.1 (64–81.4)	<0.001
Gender			<0.001
Male	704 (52.6%)	202 (65%)	
Female	634 (47.4%)	109 (35%)	
Smoking	255 (19.1%)	48 (15.4%)	0.16
Hypertension	608 (45.4%)	213 (68.5%)	<0.001
Type 2 Ddiabetes mellitus	542 (40.5%)	158 (50.8%)	0.001
Ischemic heart disease	285 (21.3%)	146 (46.9%)	<0.001
Chronic lung disease	198 (14.8%)	83 (26.7%)	<0.001
Heart failure	159 (11.9%)	76 (24.4%)	<0.001
Chronic kidney disease	137 (10.2%)	73 (23.5%)	<0.001
Cancer	114 (8.5%)	81 (26%)	<0.001
Liver disease	98 (7.3%)	17 (5.5%)	0.301
Obesity	41 (3.1%)	6 (1.9%)	0.371

**Notes.**

Values are presented as number (percentage), age is reported as median (interquartile range).

Hospitalized patients were significantly older than outpatients. A higher proportion of hospitalized patients were male. Smoking was less prevalent in hospitalized patients compared to outpatients. Comorbidities were more common among hospitalized patients, including HT, T2DM, CLD, IHD, LD, cancer, CKD, and HF. No significant difference in obesity prevalence was observed. Table 3 presents patient characteristics by hospitalization status.

**Table 3:** Patient characteristics by hospitalization status.

Variable	Outpatients (*n* = 13,326)	Hospitalized (*n* = 1,649)	*p*-value
Age (years)	38 (27.6–49.2)	58.2 (47.6–70.3)	<0.001
Gender			<0.001
Male	6,707 (50.3%)	906 (54.9%)	
Female	6,619 (49.7%)	743 (45.1%)	
Smoking	4,879 (36.6%)	303 (18.4%)	<0.001
Hypertension	1,803 (13.5%)	821 (49.8%)	<0.001
Type 2 Ddiabetes mellitus	1,897 (14.2%)	700 (42.4%)	<0.001
Chronic lung disease	1,001 (7.5%)	281 (17%)	<0.001
Ischemic heart disease	761 (5.7%)	431 (26.1%)	<0.001
Liver disease	610 (4.6%)	115 (7%)	<0.001
Cancer	432 (3.2%)	195 (11.8%)	<0.001
Chronic kidney disease	288 (2.2%)	210 (12.7%)	<0.001
Heart failure	252 (1.9%)	235 (14.3%)	<0.001
Obesity	336 (2.5%)	47 (2.9%)	0.474

**Notes.**

Values are presented as number (percentage), age is reported as median (interquartile range).

In univariable analysis, age (HR = 1.11 per year, 95% CI [1.11–1.12]), male sex, (HR = 1.66, 95% CI [1.34–2.06]) HT (HR = 9.47, 95% CI [7.61–11.79]), T2DM (HR = 5.11, 95% CI [4.16–6.29]), IHD (HR = 10.51, 95% CI [8.54–12.95]), CLD (HR = 3.59, 95% CI [2.82–4.56]), HF (HR = 10.36, 95% CI [8.13–13.20]), CKD (HR = 10.07, 95% CI [7.90–12.84]), and cancer (HR = 9.07, 95% CI [7.18–11.45]) were significantly associated with mortality. Smoking (HR = 0.363, 95% CI [0.27–0.48]) was inversely associated with mortality. LD (HR = 1.16, 95% CI [0.74–1.83]) and obesity (HR = 0.76, 95% CI [0.36–1.60]) were not significantly associated with mortality. Multivariable Cox regression identified increasing age (aHR = 1.1 per year, 95% CI [1.09–1.11]), male sex (aHR = 1.91, 95% CI [1.53–2.38]), T2DM (aHR = 1.28, 95% CI [1.01–1.61]), IHD (aHR = 1.73, 95% CI [1.36–2.21]), HF (aHR = 1.44, 95% CI [1.10–1.89]), CKD (aHR = 1.46, 95% CI [1.12–1.91]), and cancer (aHR = 2.06, 95% CI [1.60–2.65]) as significant predictors of mortality. Smoking (aHR = 0.71, 95% CI [0.53–0.95]) remained inversely associated with mortality. HT (aHR = 1.18, 95% CI [0.90–1.54]), CLD (aHR = 0.99, 95% CI [0.76–1.29]), LD (aHR = 0.69, 95% CI [0.44–1.10]), and obesity (aHR = 1.31, 95% CI [0.61–2.81]) were not significant predictors in the multivariable model (Table 4). No evidence was found for a non-linear association between age and 1-year mortality (*p* = 0.11), supporting the use of a linear age term. The attenuation from univariable to multivariable estimates—most notably for CKD—was largely explained by strong confounding from age and coexisting cardiometabolic conditions; VIFs indicated no multicollinearity concerns (Fig. S3).

**Table 4:** Univariable and multivariable analysis of risk factors associated with mortality.

	Univariable analysis			Multivariable analysis		
Variable	**HRaHR**	**95% CI**	***p*** **-value**	**aHR**	**95% CI**	***p*** **-value**
Age (years)	1.113	(1.105–1.12)	<0.001	1.096	(1.087–1.105)	<0.001
Male sex	1.66	(1.339–2.058)	<0.001	1.905	(1.527–2.375)	<0.001
Smoking	0.363	(0.274–0.481)	<0.001	0.711	(0.534–0.947)	0.02
Hypertension	9.472	(7.611–11.788)	<0.001	1.18	(0.902–1.542)	0.227
Diabetes mellitusType 2 diabetes mellitus	5.113	(4.155–6.292)	<0.001	1.276	(1.011–1.611)	0.04
Ischemic heart disease	10.513	(8.537–12.946)	<0.001	1.732	(1.359–2.207)	<0.001
Chronic Lung Disease	3.592	(2.823–4.569)	<0.001	0.99	(0.763–1.285)	0.941
Heart failure	10.359	(8.127–13.203)	<0.001	1.439	(1.096–1.891)	0.009
Chronic kidney disease	10.074	(7.904–12.84)	<0.001	1.464	(1.12–1.914)	0.005
Cancer	9.069	(7.182–11.451)	<0.001	2.06	(1.604–2.646)	<0.001
Liver Disease	1.165	(0.742–1.83)	0.506	0.694	(0.44–1.095)	0.117
Obesity	0.758	(0.359–1.602)	0.468	1.314	(0.614–2.814)	0.482

**Notes.**

HR Hazard Ratio aHR Adjusted Hazard Ratio CI Confidence Interval

Inverse probability weighting adjusted for age, sex, and comorbidities showed that current smoking was associated with a lower 360-day mortality (HR = 0.66, 95% CI [0.49–0.89]; *p* = 0.006). The *E*-value was 2.38 (lower bound: 1.49), indicating that an unmeasured confounder would need to increase both the likelihood of smoking and mortality by over two-fold to fully explain the association (Table S1).

The apparent C-index was 0.93, and after bootstrap optimism correction, it remained high at 0.89, indicating strong model discrimination (*i.e.,* ranking the mortality risk correctly in about 9 out of 10 patient pairs). For comparison, a C-index of 0.5 indicates random prediction, and 1.0 represents perfect discrimination (Harrell, Lee & Mark, 1996). The global Schoenfeld test indicated moderate non-proportionality (*χ*^2^ = 31.3 on 12 df, *p* = 0.002), primarily driven by the cancer variable (individual *p* < 0.001). Visual inspection of scaled Schoenfeld residuals showed a mild early hazard elevation for cancer patients. VIFs for all 12 covariates in the multivariable model ranged from 1.02 to 1.50, indicating no multicollinearity concerns (Table S2).

Timing of deaths was concentrated early in follow-up across major comorbidity groups. Among decedents within each condition, the proportion of deaths occurring within the first 60 days was: cancer 66.0%, T2DM 76.9%, HF 79.1%, hypertension 80.9%, IHD 76.2%, liver disease 60.0%, chronic lung disease 80.7%, obesity 71.4%, and CKD 74.4%. This descriptive timing pattern indicates that mortality among these high-risk groups is concentrated in the early post-acute period.

In the Fine–Gray subdistribution hazard model applied to hospitalized patients (*n* = 1,649), treating discharge alive as a competing event, the following were significant predictors of in-hospital mortality: each additional year of age increased the subdistribution hazard by 7%, male sex, cancer, and IHD. Smoking was not associated with in-hospital mortality. Other comorbidities—including T2DM, HF, HT, CKD, CLD, LD, and obesity—were not significantly associated with in-hospital mortality (Table S3).

## Discussion

This study analyzed a large cohort of 14,975 adults with PCR-confirmed COVID-19 and followed them for one year to investigate mortality rates and associated risk factors. The overall mortality rate was 2.4%, with 357 deaths. Age, male sex, and comorbidities such as cancer, T2DM, HF, IHD, and CKD were significantly associated with increased all-cause mortality. Each additional year of age was associated with a 10% increase in mortality risk, a finding consistent with previous studies conducted globally (Goel et al., 2021; Ioannou et al., 2020; Chatterjee et al., 2021).

Male sex was also a significant risk factor for mortality. Although some studies did not find a sex difference in COVID-19 outcomes (Ioannou et al., 2020), others, including a study based on data from 17 million patients, reported significantly higher mortality among males (Williamson et al., 2020; Günster et al., 2021). Biological differences, including immune response genes located on the X chromosome and the influence of sex hormones, likely contribute to these disparities (Klein & Flanagan, 2016; Robinson et al., 2011).

Current smokers in our cohort were younger and had fewer comorbidities at baseline. Although inverse probability of treatment weighting (IPTW) balanced measured covariates, residual confounding due to unmeasured variables such as socioeconomic status or frailty may explain the observed protective association. Prior studies have debated the so-called “smoking paradox”, with some proposing mechanisms such as nicotine’s modulation of ACE2 receptor expression (Brake et al., 2020). However, most meta-analyses in pre-vaccination cohorts have found either neutral or harmful associations with smoking (Gallus et al., 2023; Patanavanich & Glantz, 2020). Our findings, based on a stratified and weighted analysis, align with a subset of studies that suggest a protective signal, although this is likely due to selection or measurement bias.

Comorbidities played a significant role in determining outcomes (Günster et al., 2021). T2DM was the second most common comorbidity and was independently associated with mortality. T2DM related microvascular complications may exacerbate endothelial dysfunction during COVID-19, impairing oxygen delivery and tissue repair (Oguz, Koca & Yildiz, 2022). Other studies have shown that T2DM increases the risk of hospital readmission (McKay et al., 2021), mechanical ventilation (Gregg, Sophiea & Weldegiorgis, 2021), and death (Thakur et al., 2021). In a meta-analysis of 8,807 patients, T2DM was significantly associated with mortality (Chatterjee et al., 2021), and a nationwide study in Türkiye confirmed its link to adverse outcomes, including ICU admission and intubation (Guan et al., 2020).

IHD was another important predictor of mortality. Acute infections increase myocardial demand, contributing to myocardial injury or infarction. SARS-CoV-2 infection may promote atherosclerosis and thrombosis through cytokine-mediated inflammation (Bonow et al., 2020). While one study found no link between IHD and 30-day mortality (Panagiotou et al., 2021), others reported a significant association (Miller et al., 2021; Antos et al., 2021). HF also emerged as a mortality predictor. Some studies in older populations found no association between HF and in-hospital death (Görgülü & Duyan, 2020), while others documented higher mortality between 30- and 180-days post-infection (Günster et al., 2021). The observed differences likely reflect variation in follow-up durations and population characteristics.

CKD was significantly associated with 1-year mortality. Prior studies and meta-analyses have consistently shown CKD as a risk factor for poor outcomes, including respiratory failure, thromboembolic events, and death (Goel et al., 2021; Chatterjee et al., 2021; Thakur et al., 2021; Singh et al., 2022). COVID-19 may affect the kidneys through ACE2 receptors, TMPRSS2, and cathepsin L-mediated pathways, facilitating viral entry into renal tissue (Benedetti et al., 2020).

Cancer patients faced a high risk of COVID-19-related death, attributed to immunosuppression, other comorbidities, and organ dysfunction from the primary or metastatic tumor. Some cancer types confer a higher mortality risk, but the elevated risk persists regardless of cancer subtype (Cai et al., 2021; Mehta et al., 2020). In proportional-hazards diagnostics, the only covariate showing meaningful non-proportionality was cancer, with an elevated hazard concentrated in the early follow-up and attenuation thereafter. In the pre-vaccination period, patients with cancer experienced substantial early mortality following COVID-19 diagnosis. In the CCC19 cohort, deaths were concentrated within the first 30 days, reflecting a pronounced early risk window (Kuderer et al., 2020).

The association between CLD and COVID-19 outcomes remains debated. While its prevalence among COVID-19 patients is lower than expected, recent studies support its role in severe disease and mortality (Grasselli et al., 2020), although others disagree (Ioannou et al., 2020).

HT, the most common comorbidity in COVID-19 patients, was not significantly associated with mortality in our multivariable model. Although some studies identified it as a risk factor (McKay et al., 2021; Antos et al., 2021), others did not (Iaccarino et al., 2020). The attenuation likely reflects confounding by correlated cardiometabolic and organ comorbidities already included in the model, indicating that hypertension does not retain an independent association with mortality once these downstream conditions are accounted for. The absence of meaningful multicollinearity supports interpreting this reduction as clinical confounding rather than model instability. Consistent with this, prior COVID-19 cohorts have shown that the crude association between hypertension and mortality is substantially weakened after more comprehensive adjustment for age and multimorbidity (Iaccarino et al., 2020). In addition, variations in HT management and severity may explain these differences. Obesity is another controversial factor. While some studies reported no association between obesity and COVID-19-related death (Iaccarino et al., 2020), others—particularly from high-income countries—identified it as a risk factor (Williamson et al., 2020). The health impact of obesity may vary across socio-economic contexts. However, in this study interpretation of obesity constrained by low prevalence and very few events among patients, resulting in wide confidence intervals and limited power to detect moderate effects. For patients with LD, mortality rates have ranged widely. Some studies found high mortality in cirrhotic patients with COVID-19 (Iavarone et al., 2020), while others reported similar outcomes to those with cirrhosis-related complications unrelated to the virus (Bajaj et al., 2021). A Turkish cohort found no association between LD and COVID-19-related death (Görgülü & Duyan, 2020). Pooled data suggest that cirrhosis specifically, rather than general LD, is associated with increased mortality risk.

In the Turkish context, the comorbidities associated with 1-year mortality in our COVID-19 cohort parallel high baseline mortality risks in the general population. Diabetes accounts for approximately 4–5% of all deaths nationally, and ischemic heart disease remains the leading single cause of mortality, responsible for nearly one-third of deaths in Türkiye. Heart failure affects 2–3% of adults, and chronic kidney disease is prevalent in about 16% of the population, with hemodialysis patients experiencing annual mortality near 9–10% (Turkish Statistical Institute , TUIK; WHO, 2023; Onat et al., 2014; Süleymanlar et al., 2011; Asci et al., 2016). COPD and malignancy also rank among the top causes of death. These data indicate that SARS-CoV-2 infection likely amplifies rather than creates mortality risk—accelerating existing trajectories among individuals with cardiometabolic, renal, or malignant comorbidities.

Our study’s lower in-hospital mortality rate (18.9%) compared to many European studies (Günster et al., 2021; Grasselli et al., 2020) may be attributed to the younger age of our population and early Turkish national guidelines that recommended hospitalization for all patients over 50 or with comorbidities. During the first pandemic waves, hospitalization decisions in Türkiye were guided not only by clinical severity but also by perceived risk, meaning that individuals with chronic conditions such as diabetes, cardiovascular disease, or chronic kidney disease were often admitted even with mild or moderate infection. This policy may have influenced the composition of our cohort, leading to some overrepresentation of patients with pre-existing conditions and potential selection bias. Sex-disaggregated data show roughly equal COVID-19 infection rates in males and females globally (The African Population Health Research Center, 2022), consistent with our findings. However, mortality remains disproportionately higher among males, as confirmed by multiple international datasets (Williamson et al., 2020; Günster et al., 2021; Karlberg, 2004; Matsuyama et al., 2016). Strengths of our study include one of the largest single-country cohorts with a full year of follow-up. Absence of clinical severity data at the time of diagnosis is a key limitation of our study and precludes adjustment for acute disease severity. COVID-19 vaccines had not yet been introduced in Türkiye during the study period, leaving vaccination status unavailable and limiting comparability with post-vaccination cohorts. These limitations, alongside the retrospective design and single-center scope, may restrict generalizability.

## Conclusions

In this large single-center cohort of 14,975 adults with PCR-confirmed COVID-19 followed for one year after diagnosis, all-cause mortality was 2.4%, reflecting the relatively young age of the Turkish population yet confirming that excess death persists well beyond the acute phase. Increasing age, male sex, and major chronic conditions—T2DM, IHD, HF, CKD, and cancer—were all independently associated with death, while HT, CLD, obesity, and LD were not after multivariable adjustment. The inverse association between current smoking and 1-year mortality remained unexplained and may reflect residual confounding rather than biological protection. Within the first 60 days of follow-up, 66–81% of all deaths among patients with cancer, CKD, HF, IHD, T2DM, or hypertension occurred, highlighting that excess risk in these groups is concentrated in the early post-acute phase. Structured early follow-up is therefore warranted, including at least one outpatient review within the first 2–4 weeks after diagnosis. Prospective multicenter studies are needed to validate these predictors and elucidate mechanisms underlying sex disparities and the apparent smoking paradox as COVID-19 transitions to an endemic infection.

##  Supplemental Information

10.7717/peerj.21206/supp-1 Supplemental Information 1 Data set of the study

10.7717/peerj.21206/supp-2 Supplemental Information 2 Codebook for variable codings

10.7717/peerj.21206/supp-3 Supplemental Information 3 Kaplan–Meier survival curves comparing patients according to hospitalization status The cumulative survival probability of hospitalized and non-hospitalized patients during the study follow-up period

10.7717/peerj.21206/supp-4 Supplemental Information 4 Kaplan–Meier survival curves comparing patients according to comorbid conditions Each curve represents the survival probability over time for patients with or without major comorbidities.

10.7717/peerj.21206/supp-5 Supplemental Information 5 Correlation heatmap showing the relationship between comorbid conditions Pairwise correlations between the presence of major comorbidities among study participants. Color intensity represents the strength and direction of the correlation.

10.7717/peerj.21206/supp-6 Supplemental Information 6 Sensitivity Analyses for the Association Between Current Smoking and 360-Day Mortality * Abbreviations*: IPTW = inverse probability of treatment weighting. E-value quantifies the minimum strength of association that an unmeasured confounder would need with both the exposure and the outcome, beyond the measured covariates, to fully explain away the observed association (point) or to move the confidence interval to include the null (lower CI).

10.7717/peerj.21206/supp-7 Supplemental Information 7 Variance-Inflation Factors (VIFs) for Multivariable Cox Model

10.7717/peerj.21206/supp-8 Supplemental Information 8 Competing risk analysis for hospitalized patients

10.7717/peerj.21206/supp-9 Supplemental Information 9 ICD-10 codes for defining each comormid condition

## Additional Information and Declarations

### Data Availability

The following information was supplied regarding data availability: The raw data is available in the Supplemental Files.
