# Supplementary figures and images for: One-year all-cause mortality and comorbidity predictors in 14,975 adults with PCR-confirmed COVID-19: a retrospective Turkish cohort study
Source: PeerJ. 2026 Apr 20;14:e21206. doi: 10.7717/peerj.21206 (PMC13105189; doi:10.7717/peerj.21206)

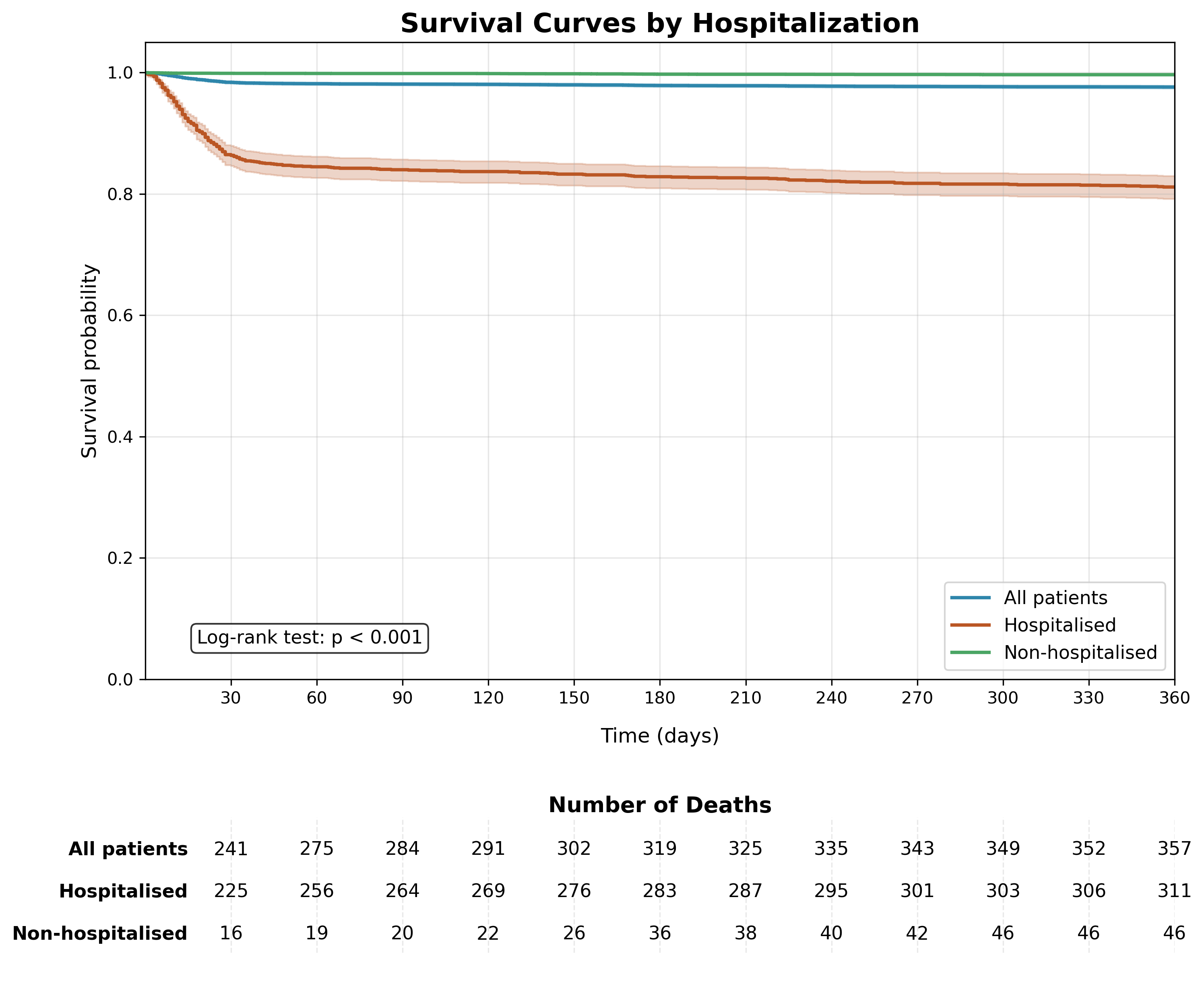

Supplement: Supplemental Information 3 — The cumulative survival probability of hospitalized and non-hospitalized patients during the study follow-up period [file peerj-14-21206-s003.png]

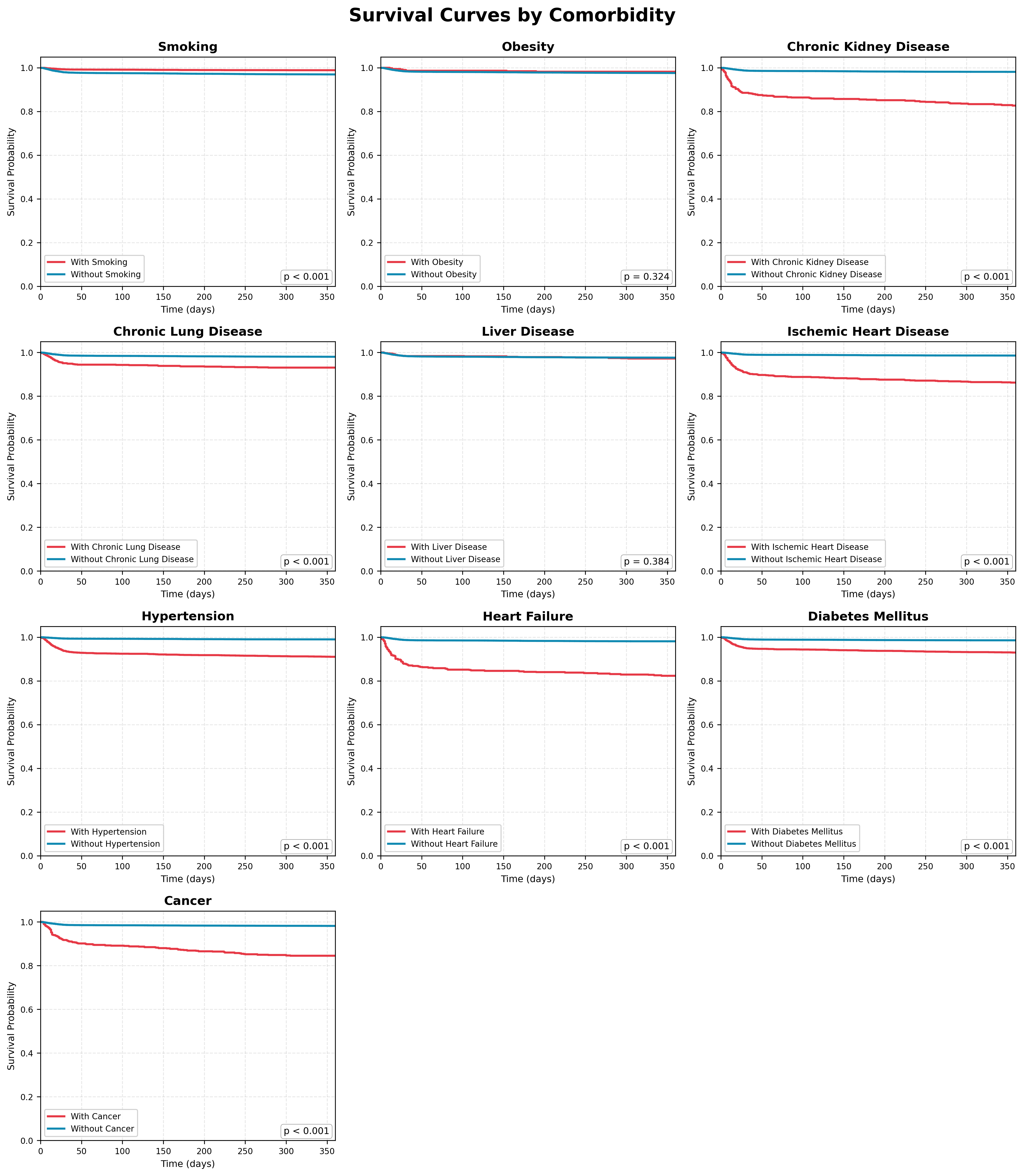

Supplement: Supplemental Information 4 — Each curve represents the survival probability over time for patients with or without major comorbidities. [file peerj-14-21206-s004.png]

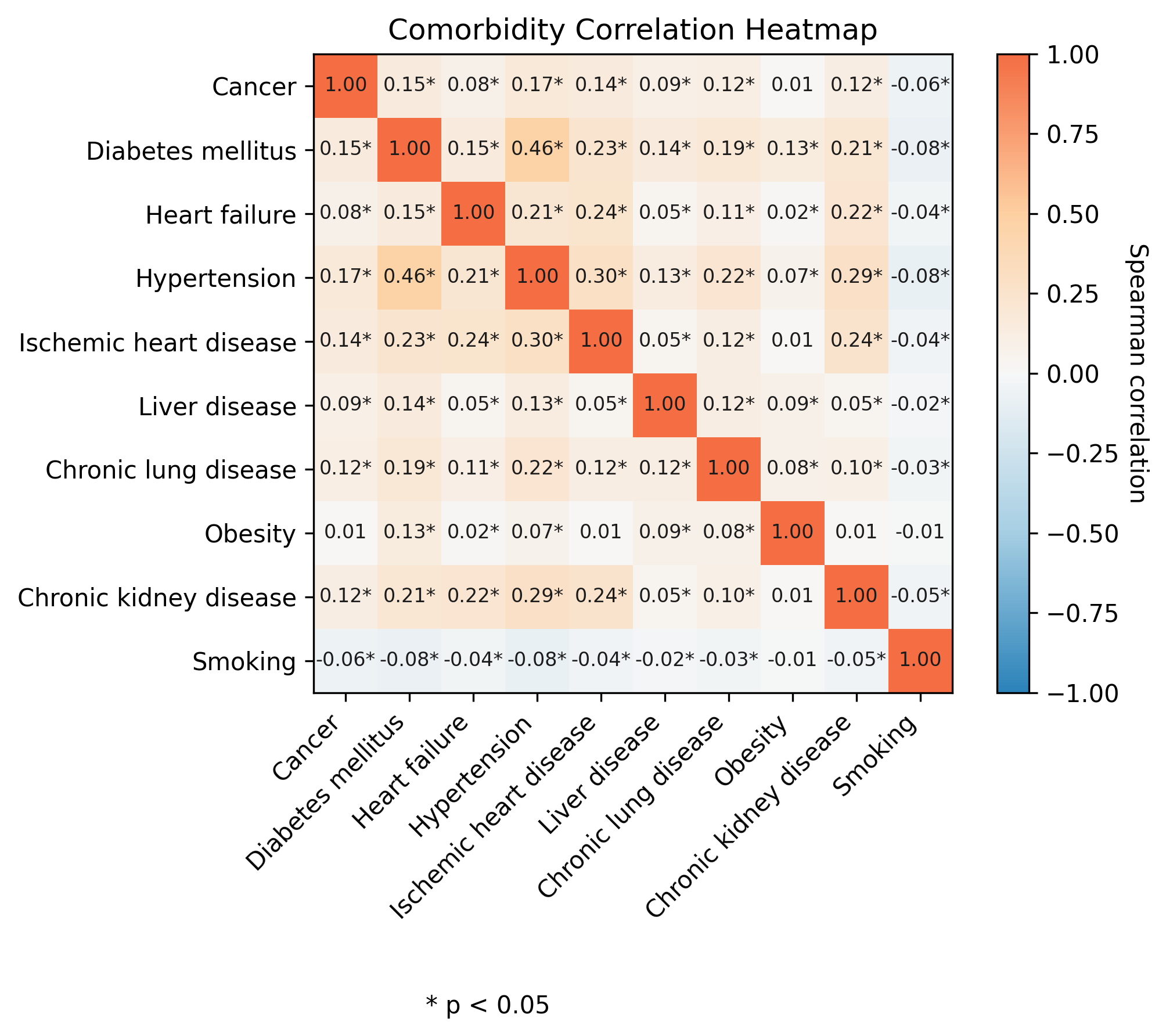

Supplement: Supplemental Information 5 — Pairwise correlations between the presence of major comorbidities among study participants. Color intensity represents the strength and direction of the correlation. [file peerj-14-21206-s005.png]
