# Supplementary material for: One-year all-cause mortality and comorbidity predictors in 14,975 adults with PCR-confirmed COVID-19: a retrospective Turkish cohort study
Source: PeerJ. 2026 Apr 20;14:e21206. doi: 10.7717/peerj.21206 (PMC13105189; doi:10.7717/peerj.21206)
Supplement: Supplemental Information 6 — Abbreviations: IPTW = inverse probability of treatment weighting. E-value quantifies the minimum strength of association that an unmeasured confounder would need with both the exposure and the outcome, beyond the measured covariates, to fully explain away the observed association (point) or to move the confidence interval to include the null (lower CI). [file peerj-14-21206-s006.docx]

**Supplementary Table S1. Sensitivity Analyses for the Association Between Current Smoking and 360‑Day Mortality**

| Analysis approach | Hazard ratio (95 % CI) | *p*-value | E‑value (point) | E‑value (lower CI) |
| --- | --- | --- | --- | --- |
| IPTW (main model) | 0.66 (0.49-0.89) | 0.006 | 2.38 | 1.49 |
| IPTW, weights truncated at 5th/95th pct | 0.68 (0.50-0.92) | 0.010 | 2.17 | 1.46 |
| Doubly robust (IPTW + outcome regression) | 0.70 (0.52-0.94) | 0.020 | 2.01 | 1.45 |

***Abbreviations*: IPTW =** inverse probability of treatment weighting. E‑value quantifies the minimum strength of association that an unmeasured confounder would need with both the exposure and the outcome, beyond the measured covariates, to fully explain away the observed association (point) or to move the confidence interval to include the null (lower CI).
