# Supplementary material for: One-year all-cause mortality and comorbidity predictors in 14,975 adults with PCR-confirmed COVID-19: a retrospective Turkish cohort study
Source: PeerJ. 2026 Apr 20;14:e21206. doi: 10.7717/peerj.21206 (PMC13105189; doi:10.7717/peerj.21206)
Supplement: Supplemental Information 7 [file peerj-14-21206-s007.docx]

**Supplementary Table S2. Variance‑Inflation Factors (VIFs) for Multivariable Cox Model**

| Covariate | VIF |
| --- | --- |
| Age (years) | 1.35 |
| Male Sex | 1.06 |
| Smoking | 1.04 |
| Hypertension | 1.5 |
| Type 2 diabetes mellitus | 1.26 |
| Ischemic heart disease | 1.36 |
| Chronic lung disease | 1.17 |
| Heart failure | 1.26 |
| Chronic kidney disease | 1.22 |
| Cancer | 1.15 |
| Liver Disease | 1.02 |
| Obesity | 1.04 |
