# Supplementary material for: One-year all-cause mortality and comorbidity predictors in 14,975 adults with PCR-confirmed COVID-19: a retrospective Turkish cohort study
Source: PeerJ. 2026 Apr 20;14:e21206. doi: 10.7717/peerj.21206 (PMC13105189; doi:10.7717/peerj.21206)
Supplement: Supplemental Information 8 [file peerj-14-21206-s008.docx]

**Supplementary Table S3 competing risk analysis for hospitalized patients**

| Variable | Subdistribution HR (sHR) | 95% CI | *p*-value |
| --- | --- | --- | --- |
| Age (years) | 1.065 | 1.054-1.080 | <0.001 |
| Male Sex | 1.773 | 1.398-2.250 | <0.001 |
| Smoking | 0.884 | 0.651-1.200 | 0.43 |
| Hypertension | 0.940 | 0.707-1.250 | 0.67 |
| Type 2 diabetes mellitus | 1.042 | 0.820-1.320 | 0.74 |
| Ischemic heart disease | 1.335 | 1.043-1.710 | 0.022 |
| Chronic lung disease | 1.179 | 0.894-1.550 | 0.24 |
| Heart failure | 1.100 | 0.828-1.460 | 0.51 |
| Chronic kidney disease | 1.151 | 0.856-1.550 | 0.35 |
| Cancer | 1.878 | 1.427-2.470 | <0.001 |
| Liver Disease | 0.746 | 0.463-1.200 | 0.23 |
| Obesity | 1.380 | 0.600-3.170 | 0.45 |
