# Supplementary material for: One-year all-cause mortality and comorbidity predictors in 14,975 adults with PCR-confirmed COVID-19: a retrospective Turkish cohort study
Source: PeerJ. 2026 Apr 20;14:e21206. doi: 10.7717/peerj.21206 (PMC13105189; doi:10.7717/peerj.21206)
Supplement: Supplemental Information 9 [file peerj-14-21206-s009.docx]

**Supplementary Table S4. Definitions of comorbidities and smoking status**

| Variable | ICD-10 codes (if applicable) |
| --- | --- |
| Hypertension | I10–I15 |
| Type 2 diabetes mellitus | E10–E14 |
| Ischemic heart disease | I20–I25 |
| Heart failure | I50 |
| Chronic kidney disease | N18–N19 |
| Chronic lung disease | J40–J47, J60–J67 |
| Liver disease | K70–K77 |
| Cancer | C00–C97 |
| Obesity | E66 |
| Smoking status | “Active smoking” = current daily or occasional use; former smoking not available |
